# Single intravenous dose ondansetron induces QT prolongation in adult emergency department patients: a prospective observational study

**DOI:** 10.1186/s12245-024-00621-5

**Published:** 2024-04-02

**Authors:** Mohammad Rezaei Zadeh Rukerd, Fatemeh Rafiei Shahrbabaki, Mitra Movahedi, Amin Honarmand, Pouria Pourzand, Amirhossein Mirafzal

**Affiliations:** 1 Gastroenterology and Hepatology Research Center, Institute of Basic and Clinical Physiology Sciences, Kerman University of Medical Sciences, Kerman, Iran; 2 Department of Emergency Medicine, Kerman University of Medical Sciences, Kerman, Iran

**Keywords:** Ondansetron, QT prolongation, Prediction, Emergency department

## Abstract

**Background:**

Ondansetron is one of the most commonly used drugs in the emergency department (ED) for treating nausea and vomiting, particularly in intravenous (IV) form. Nevertheless, it has been shown to prolong QT interval and increase the risk of ventricular dysrhythmias. This study evaluated the associations between single IV ondansetron dosage and subsequent QTc prolongation in the ED.

**Methods:**

In this prospective observational study, a total number of 106 patients presenting to the ED in a 3-month period with nausea and vomiting treated with IV ondansetron were enrolled. QT and QTc intervals were measured at baseline (QT0 and QTc0), and 60 min (QT60 and QTc60) following a single-dose administration of ondansetron at 4 or 8 mg doses. To evaluate the predictive ability of these variables, we employed receiver operating characteristic (ROC) curve analyses.

**Results:**

The predictive models for QTc prolongation 1-hour post-ondansetron administration showed the following: at baseline, the area under curve of 0.70 for QT, 0.71 for QTc, and 0.64 for dosage. Conversely, a QTc0 = 375 msec indicated a QTc60 > 480 msec with a specificity of 97%. Additionally, a QTc0 of 400 msec had a sensitivity of 100% in predicting a QTc60 < 480 msec, while a QTc0 > 460 msec predicted a QTc60 > 480 msec with a specificity of 98%. Moreover, 8 mg doses were associated with higher rates of QTc60 prolongation, while 4 mg doses favored maintaining QTc60 within normal limits.

**Conclusions:**

Our study demonstrates the predictive capacity of QT0, QTc0, and ondansetron dosage in forecasting QTc60 prolongation (> 480 msec) post-ondansetron administration. These findings advocate for their incorporation into clinical protocols to enhance safety monitoring in adult ED patients.

## Background

Sudden Cardiac Death (SCD), one of the most challenging public health issues, is primarily caused by arrhythmias secondary to structural heart disease or primary electrical heart abnormalities [1–4]. While several factors can lead to arrhythmias, one of the most important of which is the prolonged QT interval on the electrocardiogram (ECG) [5, 6]. The QT interval duration depends on variety of factors, for which various formulas are described to interpret the corrected QT intervals (QTc) [7–10]. Most guidelines recommend QTc > 450, QTc > 480, and QTc > 500 msec as the absolute QTc prolongation based on the severity [7, 9, 11–13]. Several factors are associated with prolonged QTc, such as electrolyte imbalance and medications, particularly as a result of drug-drug interactions or the intake of QT prolonging drugs [9, 14, 15].

Numerous medications can potentially cause this complication, including various antiarrhythmics, antibacterials, antifungals, opioids, antipsychotics, antidepressants, and antiemetics [16]. Among antiemetics, ondansetron has been utilized to minimize postoperative, chemotherapy and radiation-induced nausea and vomiting [17]. Ondansetron is considered one of the most commonly used drugs in the emergency department (ED) since it has fewer adverse effects and similar efficacy as other antiemetics, such as metoclopramide and promethazine [18, 19]. Nevertheless, concerns remain regarding the possibility of prolonging the QTc interval and increasing the risk of ventricular dysrhythmias [20]. In pediatrics, intravenous (IV) ondansetron did not cause QTc prolongation in the emergency department [21, 22]. Studies have shown that a single 4 mg IV ondansetron injection can increase the QTc interval in adult ED patients [23, 24]. However, the findings across studies are conflicting. Most of these studies focused on patients who already had a prolonged QTc at the start, while only a few have examined whether this increase is related to the dose given and what potential clinical effects it might have [25, 26].

In this study, we aimed to further investigate the impact of baseline QT/QTc measurements and IV ondansetron dosage on the QTc interval prolongation in adult ED patients without long-QT risk factors. Our findings can help emergency physicians understand better when to use ondansetron as a first-line treatment in the ED.

## Methods

### Study design

The study protocol was in line with human subject protection regulations, approved by the Research Ethics Committee of Kerman Medical University (IR.KMU.AH.REC.1399.007), and written informed consent was obtained from each participant.

### Study population and setting

In this prospective observational study, the inclusion criteria consisted of all adult patients referred to the ED of the Bahonar Hospital (an academic referral hospital in Kerman, Iran) between October 2021 and January 2022, who experienced nausea and vomiting during hospitalization and were treated with intravenous ondansetron at the discretion of the treating physician. Patients were excluded if they had a history of taking drugs that are known to cause QTc prolongation before admission or during the ED stay (e.g. macrolides, fluoroquinolones, antifungals, antipsychotics, antidepressants or methadone), had hypokalemia or hypocalcemia, had a baseline ECG abnormality such as hemiblocks or bundle branch blocks, left the emergency department less than 2 h after admission (due to inability to get an ECG after ondansetron administration), or declined to participate in the study.

### Study protocol, variables and outcomes

All 12-lead ECGs in this study were obtained using a Dena650 ECG device (Saadatco, Tehran, Iran). QTc intervals were measured and calculated by two trained EPs blinded to the intent of the study, using Bazett Formula (QTcB = QT/RR^1/2), and reported as milliseconds (msec). The degree of agreement (kappa coefficient) between the two evaluators was measured. Any discrepancies between the two EPs were resolved through consultation with a third EP.

Each patient had a 12-lead ECG taken before receiving ondansetron and again at 60 minutes after the injection. The initial QT and QTc interval (QT0 and QTc0), and QT and QTc interval at 60-minutes post infusion (QT60 and QTc60) were calculated. The quantity of ondansetron injected for each patient, a dose of 4 or 8 mg, was recorded. Additionally, other variables such as demographic characteristics, initial vital signs, past medical history (e.g. diabetes mellitus or chronic obstructive pulmonary disease), and opium use (ascertained through patients’ self-report) were included. The primary outcome of our study was evaluation of QTc > 480 msec at one-hour post-injection. Furthermore, the correlation between IV ondansetron dose and the subsequent prolongation in QTc interval was also studied as our secondary outcome.

### Sample size and statistical analyses

According to the formula of sample size calculation for diagnostic tests, considering respective type I and II errors of 5 and 20%, and with regard to the α and β values in a relatively similar study [23], a minimum sample size of 30 (15 for each group: QTc ≤ 480 msec vs. QTc > 480 msec) was calculated.

Quantitative variables with normal and non-normal distribution were described as mean (SD) and median (interquartile range), respectively, while for qualitative variables, percent of frequency was used. t-test and chi-square were applied to assess the associations of continuous and categorical variables between the two groups (with normal distribution), respectively. The degree of associations was expressed by odds ratio (OR) and 95% confidence interval (CI). Lastly, the receiver operating characteristics (ROC) curve and Youden’s index were implemented for determining the best cut-off point holding the highest simultaneous sensitivity and specificity for the prediction of the outcomes.

A p-value of less than 0.05 was considered statistically significant in all statistical tests. Statistical analyses were performed using SPSS version 16.0 (SPSS Inc., Chicago, IL, USA).

## Results

**Fig. 1:**
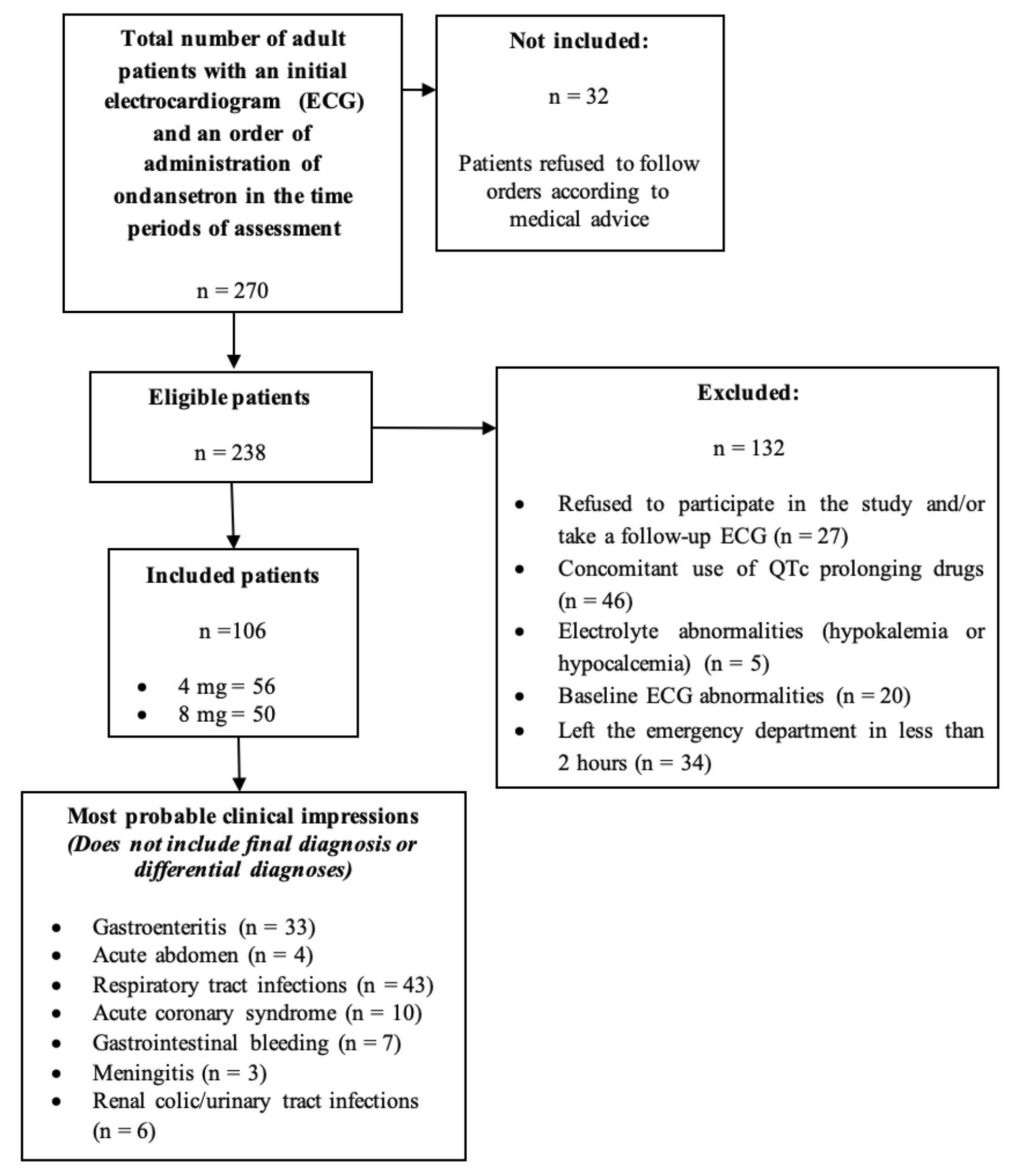
Enrollment procedure flow diagram, comprising of inclusion and exclusion criteria

A total of 106 patients were enrolled in the study (Fig. 1). Forty-eight (45.3%) were males and fifty-eight (54.7%) were females. Fifty-six patients (52.8%) received 4 mg and fifty (47.2%) received 8 mg. Twenty-two (20.8%) patients reported opium use, whereas 14 (13.2%) had a history of co-morbidities such as diabetes mellitus, non-complicated hypertension and chronic obstructive pulmonary disease. The mean (SD) value for initial QTc interval was 414 (21) msec with a minimum and maximum of 364 and 457 msec, respectively (Table 1).

**Table 1:** Basic characteristics of quantitative variables

		Age (years)	HR (beats/min)	SBP (mmHg)	QT0 (msec)	QTc0 (msec)
Total	Mean (SD) Minimum Maximum	48.1 (15.4) 19 88	93.4 (12) 68 120	123.6 (16.4) 100 190	333.9 (26.4) 270 387	414.3 (24.4) 364 457
Received dose of 4 mg ondansetron	Mean (SD) Minimum Maximum	50.8 (17.6) 19 88	94.4 (10.8) 78 120	128.8 (20.7) 100 190	331.3 (25.3) 270 387	414.1 (25.8) 364 457
Received dose of 8 mg ondansetron	Mean (SD)	44.8 (12.5)	92.2 (13.3)	117.9 (6.4)	336.6 (27.9)	414.6 (23.3)
	Minimum	85	68	110	277	368
	Maximum	25	120	140	378	457

HR: Initial heart rate, SBP: Initial systolic blood pressure, QT0: Baseline QT interval before ondansetron administration, QTc0: Baseline QTc interval before ondansetron administration,

QTc interval at 60 min following the administration of ondansetron were longer. The mean (SD) value for the increase in QTc interval 60-minutes following administration were 54.7 (25.1) msec, with increments from the baseline (QTc0) to QTc60 being statistically significant (*p* < 0.001). While none of the patients had a QTc interval longer than 480 msec before the administration, 28 (26.4%) showed QTc intervals over 480 msec (prolonged QTc) after 60 min. The only variables which showed associations with prolonged QT at 1-hour post-ondansetron administration were QT0, QTc0, and ondansetron dose (Tables 2 and 3). The sensitivity and specificity of QT0 and QTc0 at various cutoff values for predicting QTc60 > 480 msec are presented in Table 4.

**Table 2:** Association of quantitative variables with QTc intervals over 480 msec at 60 min following ondansetron administration

		Age (years)	HR (beats/min)	SBP (mmHg)	QT0 (msec)	QTc0 (msec)
Total	QTc60 ≤ 480 msec	49.2 (16.9)*	93.9 (13.5)	125.3 (18.4)	328.8 (27.3)	408.8 (24.4)
	QTc60 > 480 msec	46.1 (12.4)	93.7 (9.6)	120.2 (11.3)	343.8 (18.4)	424.6 (20.7)
	P value	0.46	0.71	0.24	0.03**	0.01**

HR: Initial heart rate, SBP: Initial systolic blood pressure, QTc 0: QTc interval before the administration of ondansetron

*All values are shown in mean (SD)

**Statistical significance

**Table 3:** Association of qualitative variables with QTc intervals over 480 msec at 60 min following ondansetron administration

	Geriatric (age > 65)		Gender		Past medical history †		Opium use		Ondansetron dose	
	Yes	No	Male	Female	Yes	No	Yes	No	4 mg	8 mg
QTc60 ≤ 480 msec	22*	56	32	46	10	68	14	64	48 (86%)	30 (60%)
QTc60 > 480 msec	6	22	16	12	4	24	8	20	8 (14%)	20 (40%)
P value	0.21		0.14		0.67		0.35		0.04*	

†Past medical history: Diabetes mellitus and chronic obstructive pulmonary disease

*All values are number of patients in each group

**Statistical significance

**Table 4:** Sensitivity and specificty of QT0 and QTc0 in different cutoffs for the prediction of QTc60 > 480msec

	QT0 (msec)			QTc0 (msec)		
	312	325	375	400	410	460
Sensitivity (%)	100	93	7	100	86	7
Specificity(%)	32	56	97	48	63	98

**Fig. 2:**
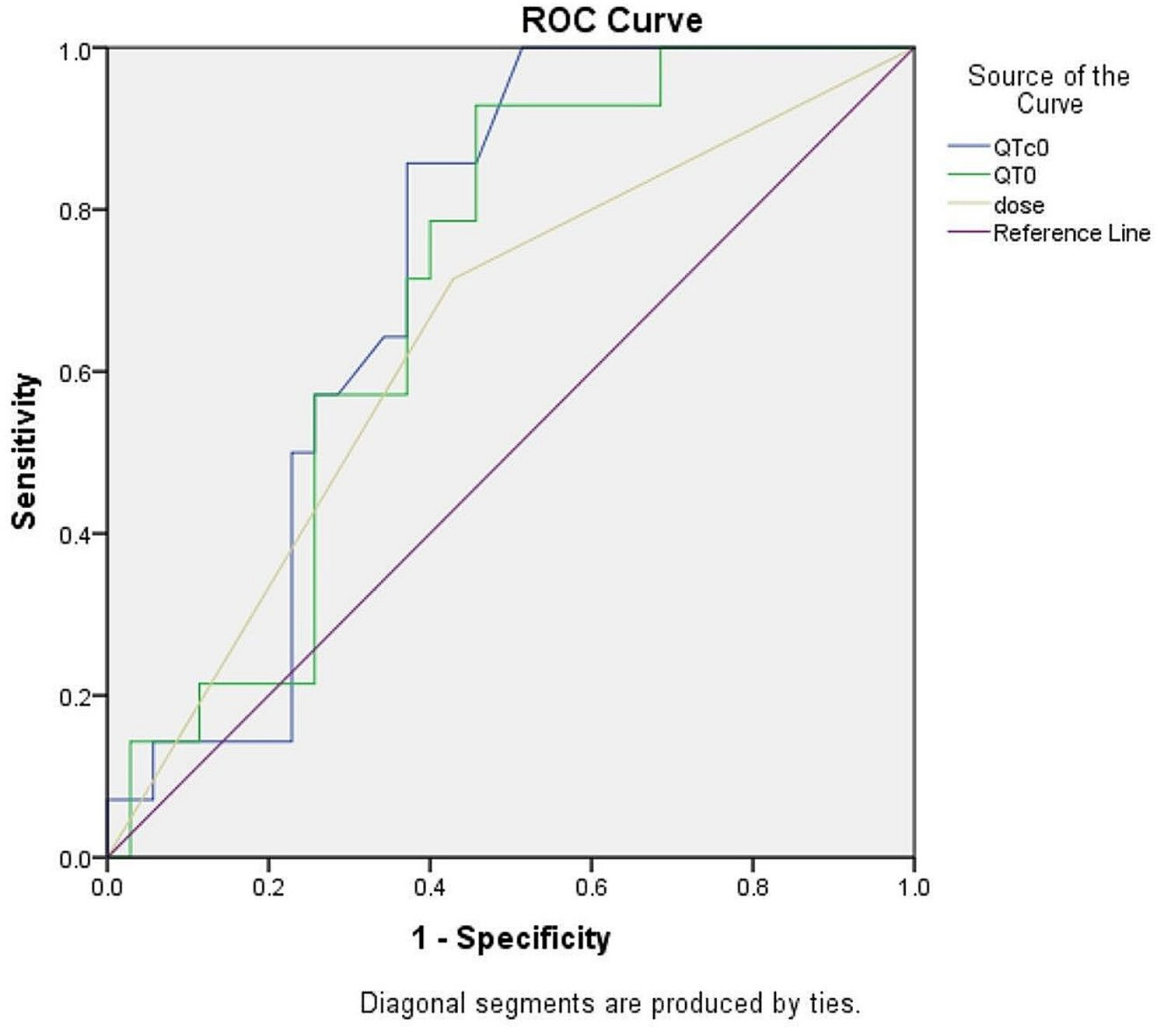
Receiver operating characteristic curve (ROC), estimating QTc prolongation 60 min following IV ondansetron administration

The ROC curve for prediction of prolonged QTc60 showed the area under curves (AUC) (95% CI) of 0.71 (0.61–0.81), 0.70 (0.55–0.84), and 0.64 (0.52–0.76) for QTc0, QT0, and ondansetron dose, respectively (Fig. 2).

Of note, no patient experienced any adverse outcomes or noticeable ventricular arrhythmias linked to QTc prolongation during the study period in the ED.

## Discussion

Based on our study, ondansetron, as a regularly used antiemetic in adult patients presenting with nausea and vomiting to the ED, carries a considerable risk for QTc prolongation even with a single dose and in those without any long-QT risk factors. Our findings demonstrated that baseline QT/QTc measurements and IV ondansetron dosage are significant predictors of QTc interval prolongation (> 480 msec) following a single IV dose of ondansetron, emphasizing the need for careful consideration of these factors as well as the balance between the risk of QTc prolongation and ventricular arrhythmias and the therapeutic benefits.

The Food and Drug Administration (FDA) has revealed that drugs that increase mean QT/QTc by more than 20 milliseconds can increase the incidence of various arrhythmias [27]. In 2011, the FDA issued a warning that intravenous ondansetron can potentially lead to fatal arrhythmias in people with prolonged QT intervals [28]. Obtaining a screening baseline ECG in patients without high-risk background has not been suggested to date, although some authors recommend screening before using oral ondansetron in high-risk patients or for those receiving an IV dose [29, 30]. Notably, while according to the current literature, the clinical impact and consequences of QTc prolongation following a single dose of IV ondansetron is under question, practitioners should be cautious about using this antiemetic in the IV form, especially for those with cardiovascular histories, known electrolyte abnormalities, and consumption of concomitant potentially QTc prolonging drugs [23].

Among pediatric population, no substantial QTc prolongation following administration of standard doses of ondansetron (0.15 mg/kg) has been observed in several studies, and the risk of ventricular dysrhythmias has been estimated to be 3 in 100,000 [21, 31]. Among adults, several studies have reported dysrhythmias and QTc prolongation following IV ondansetron administration [29, 32–35]. The most frequent dose studied was a single 4 mg IV dose, and the range of reported QTc prolongation was from 1.6 to more than 30 msec, which were observed as soon as a few minutes following administration and lasted for several hours after each dose [23, 24]. According to the results obtained from previous studies, a single IV dose of ondansetron greater than 16 mg has not been recommended [35]. Nevertheless, the results of these studies are not consistent and few of them studied patients with normal QTc at baseline, which is not always the case in clinical practice. In previous ED studies, most patients received only 4 mg of IV ondansetron, and the effect of higher doses (such as 8 mg) on QTc in adults ED patients is undetermined [23, 24]. Moreover, while some authors have demonstrated the dose-dependent nature of QTc prolongation with IV ondansetron, the literature still lacks sufficient information in the ED setting to establish clear cutoffs and recommendations for its IV use, especially for patients without known long-QT risk factors [25].

Our study could not find any connection between prolonging QTc60 and patients’ demographic or background factors. This means that when deciding whether to give ondansetron to patients who do not seem to have a high risk of QTc prolongation, we mainly need to consider their baseline QT and QTc intervals, along with the ondansetron dosage. The high specificity of baseline QTc > 460 msec for prediction of prolonged QTc60 indicates that there may be an indication for ECG screening before ondansetron administration, even in patients without any evident risk factors. However, only 10 (9.4%) showed QTc0 of more than 450 msec. Consequently, since many patients at the ED stay are routinely evaluated by an initial ECG, the QTc interval should be noticed by practitioners before ondansetron administration. On the other side, based on our results and findings of the other studies which measured QTc intervals more than 60 min following the IV dose, a QT0 of 312 msec and a QTc0 of 400 msec or less may be considered safe for IV doses of 8 mg or less in the absence of other risk factors. The AUC for ROC curve shows a good prediction capability for QTc0 and QT0, and a poor capability for ondansetron dose. However, there are only two doses in our evaluation, and widening the dose range may strengthen the predictive ability of this variable.

## Limitations

Like many studies in this field, our research had some limitations that should be acknowledged. Firstly, the limited time and resources which lead to only two measurements following the administration of the drug. Further, we had multiple exclusion criteria, but it was considered as a measure to assess the QT prolongation effect of ondansetron, mitigating other potential confounding factors. Also, in line with clinical practice, most of our patients were on pulse oximetry monitoring but not continuous ECG monitoring to assess the occurrence of any transient dysrhythmias, no clinically evident adverse effects or dysrhythmia were observed in our study. Lastly, we did not follow the patients after their disposition from the ED to evaluate their final outcome and relevance of any adverse outcome to the probable QTc prolongation. Thus, these limitations should be considered in interpreting our results and for future studies.

## Conclusion

Our study highlights the predictive value of baseline QTc (QTc0) measurements and ondansetron dosage for identifying the risk of QTc prolongation (> 480 msec) an hour after administration. It suggests that baseline QT intervals of 312 msec or less and QTc intervals of 400 ms or less may be safe thresholds for administering IV ondansetron doses up to 8 mg in patients without additional risk factors. These findings advocate for their incorporation into clinical protocols to enhance safety monitoring in adult ED patients.

## Data Availability

The corresponding author can provide supporting data for this study upon reasonable request.

## Abbreviations

SCD: Sudden cardiac death
VF: Ventricular fibrillation
ECG: Electrocardiogram
QTc: corrected QT intervals
FDA: Food and drug administration
ED: Emergency department
IV: Intravenous
EP: Emergency physician
OR: Odds ratio
CI: Confidence interval
ROC: Receiver operating characteristics
AUC: Area under curves
